# Review of Vaccination Recommendations in Guidelines for Non-Communicable Diseases with Highest Global Disease Burden among Adults 75 Years Old and Above

**DOI:** 10.3390/vaccines11061076

**Published:** 2023-06-08

**Authors:** Abdul Rahman Ishak, Yu Chun Hsieh, Harshitha Srinivasan, Kay Choong See

**Affiliations:** Department of Medicine, Yong Loo Lin School of Medicine, National University of Singapore, Singapore 117597, Singapore; e0501186@u.nus.edu (A.R.I.); e0408893@u.nus.edu (H.S.)

**Keywords:** vaccination, guidelines, geriatric, elderly, public health, review

## Abstract

This scientific review paper explores international and country-specific healthcare guidelines for non-communicable diseases with the highest burden among individuals aged 75 years and above. The study aims to identify the best vaccination practices and standardize healthcare practices to improve vaccination adherence in this vulnerable population. Given that older people are more prone to infectious illnesses and have higher rates of morbidity and mortality, vaccinations are essential for disease prevention. Despite the proven efficacy of vaccinations, adherence has plateaued in recent years, partly due to a lack of accessibility, public education, and variability in disease-specific guidelines. This paper highlights the need for a more robust and standardized international vaccination model to improve quality of life and reduce disability-adjusted life years among the elderly. The findings of this study call for further research to review the guidelines as more implementations are put in place, including non-English guidelines.

## 1. Introduction

Development of vaccinations has been touted as one of the most cost-effective and efficacious public health interventions in the world. An estimated 2.5 million deaths are prevented by vaccinations each year [1]. Increased accessibility to vaccines is prolonging life expectancy and decreasing morbidity and mortality caused by vaccine-preventable diseases. Primary prevention through vaccinations has an integral part to play in caring for patients, especially in vulnerable populations such as the elderly. There is a notable direct relationship between increasing age and susceptibility to infections among the elderly, with infectious diseases accounting for one third of deaths in adults aged 65 and older [2,3].

Our project focuses on non-communicable diseases (NCD) which are defined by the Pan American Health Organization (PAHO) as a “group of conditions that are not mainly caused by an acute infection, result in long-term health consequences and often create a need for long-term treatment and care” [4]. According to the World Health Organization (WHO), NCDs are the leading cause of death worldwide, responsible for 71% of the total number of deaths each year [5].

Vaccination adherence has plateaued in recent years, largely due to the COVID-19 pandemic and its associated disruptions [6]. Other factors such as a lack of accessibility and public education also contribute to vaccination uptake being suboptimal.

One way to shape policy and allow for better vaccine implementation is publishing guideline recommendations. The WHO has a database with papers recommending vaccinations to prevent various life-threatening conditions [7].

Disease-specific guidelines are more relevant and influential in clinical practice. However, their vaccination recommendations may be variable, contributing to suboptimal vaccination practices. Therefore, there is a need to review the healthcare guidelines of different countries, identify the role of vaccinations in the proposed management, and standardise these healthcare practices in order to boost vaccination adherence.

This project aims to examine international and country-specific healthcare guidelines on non-communicable diseases with greatest burden as quantified by mortality, morbidity, and disability-adjusted life years among the age group of 75 years old and above. With the increasing vulnerability of the older age group, this study is pertinent to identify the best vaccination practices for a more robust and standardised international vaccination model to be established.

## 2. Materials and Methods

**Figure 1:**
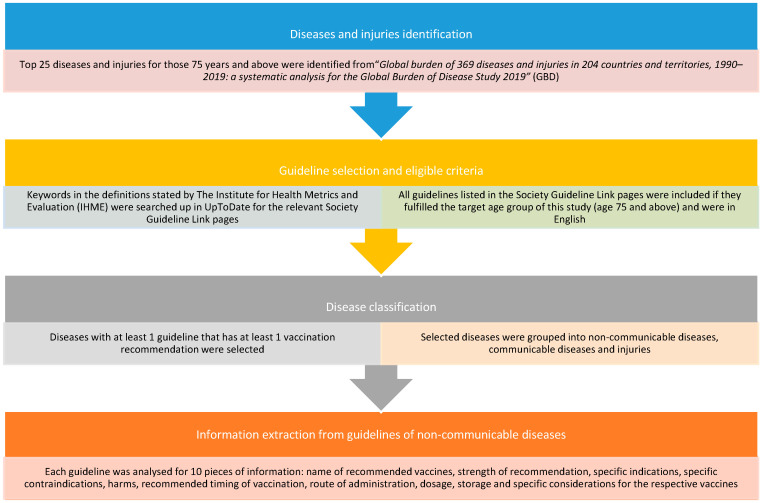
Flowchart of materials and methods.

Please see Figure 1 for a summary of the Materials and Methods.

### 2.1. Diseases and Injuries Identification

The study “Global burden of 369 diseases and injuries in 204 countries and territories, 1990–2019: a systematic analysis for the Global Burden of Disease Study 2019” (GBD) [8], was referenced for this study. The top 25 diseases and injuries for those 75 years and above, ranked according to disability-adjusted life years (DALYs), were identified.

The 25 identified diseases and injuries are as follows: ischaemic heart disease (IHD); stroke; chronic obstructive pulmonary disease (COPD); Alzheimer’s disease and other dementias; diabetes; lower respiratory infections (LRI); tracheal, bronchus, and lung cancer; falls; chronic kidney disease (CKD); age-related hearing loss; hypertensive heart disease; diarrhoeal diseases; low back pain; colon and rectal cancer; blindness and vision loss; atrial fibrillation and flutter; stomach cancer; prostate cancer, cirrhosis and other chronic liver diseases; Parkinson’s disease; osteoarthritis; oral disorders; tuberculosis (TB); asthma; and road injuries.

### 2.2. Guideline Selection and Eligible Criteria

The Institute for Health Metrics and Evaluation (IHME), which published GBD, has a definition for every disease and injury. The keywords mentioned in the definition were then searched in UpToDate for the relevant Society Guideline Link pages.

UpToDate (UpToDate Inc, Waltham, MA, USA) is an evidence-based point of care medical resource that is widely used by healthcare professionals [9] due to its feasibility and usefulness in clinical decision-making [10]; it was referenced for the list of guidelines.

Guidelines listed in the Society Guideline Link pages were included if they fulfilled the target age group of this study (age 75 and above) and were in English. The guideline selection process was undertaken from September 2022 to November 2022.

### 2.3. Disease Classification

Diseases with at least one guideline that has at least one vaccination recommendation were selected. This group of diseases was then classified into non-communicable diseases, communicable diseases and injuries. This classification system is used by WHO to analyse the composition of DALYs in the World Health Statistics 2023 [11]. 

### 2.4. Information Extraction

Each guideline of each non-communicable disease was further analysed for ten pieces of information: name of recommended vaccines, strength of recommendation, specific indications, specific contraindications, harms, recommended timing of vaccination, route of administration, dosage, storage, and specific considerations for the respective vaccines.

Strength of recommendation refers to the extent of the confidence the panel of experts have in a specific recommendation, usually following the analysis of the benefits, risks, the context, and the quality of the evidence [12]. The strength of recommendation influences the ease and/or complexity of adopting and implementing a recommendation [13].

Indications, contraindications, harms are crucial for healthcare professionals to weigh the risks and benefits of vaccination, as these affect their confidence in offering the vaccination to their patients [14,15].

## 3. Results

Out of the 25 identified diseases, 23 (ischaemic heart disease; stroke; chronic obstructive pulmonary disease; Alzheimer’s disease and other dementias; diabetes; lower respiratory infections; tracheal, bronchus, and lung cancer; falls; chronic kidney disease; age-related hearing loss; hypertensive heart disease; diarrhoeal diseases; low back pain; colon and rectal cancer; atrial fibrillation and flutter; stomach cancer; prostate cancer; cirrhosis and other chronic liver diseases; Parkinson’s disease; osteoarthritis; tuberculosis; asthma; and road injuries) contained guidelines listed in the Society Guideline Link on UpToDate. Upon further analysis of these 23 diseases, 15 (ischaemic heart disease; stroke; chronic obstructive pulmonary disease; diabetes; lower respiratory infections; falls; chronic kidney disease; hypertensive heart disease, diarrhoeal diseases; colon and rectal cancer; atrial fibrillation and flutter; stomach cancer; cirrhosis and other chronic liver diseases; tuberculosis; asthma) contained guidelines which mentioned vaccine recommendations.

Out of these 15 diseases, we will focus on 11 of the non-communicable diseases namely: ischaemic heart disease; stroke; chronic obstructive pulmonary disease; diabetes; chronic kidney disease; hypertensive heart disease; colon and rectal cancer; atrial fibrillation and flutter; stomach cancer; cirrhosis and other chronic liver diseases; and asthma). Lower respiratory infections, diarrhoeal diseases, and tuberculosis are excluded as they are communicable diseases. Falls are excluded because according to the World Health Organisation (WHO), they are grouped separately.

### 3.1. IHD

For ischaemic heart disease, 6 out of 102 guidelines mentioned vaccine recommendations. These guidelines were from the United States, Europe and Australia–New Zealand. All six guidelines recommended the influenza vaccine. Only one guideline, which was from Australia–New Zealand, recommended the pneumococcal vaccine.

This paragraph focuses on guidelines recommending the influenza vaccine. In terms of their strength of recommendation, three guidelines were strong, while three were not stated. Additionally, specific indications from the six guidelines include stable ischaemic heart disease patients aged 65 years old and above; all CABG patients unless contraindications exist; chronic coronary syndrome patients aged 65 and above; and everyone with coronary heart disease unless contraindicated. Only one guideline mentioned specific harms, which were pain and myalgia at the injection site. Overall, it is recommended that the influenza vaccine be given annually via intramuscular route, using a standard dose (volume was not mentioned). There was also no mention of any storage information. 

This paragraph focuses on the one guideline recommending the pneumococcal vaccine. The strength of recommendation, specific contraindication and harms, vaccine administration, and storage details were not mentioned. Specific indications were for everyone with coronary heart disease, unless contraindicated. A summary of the above vaccine recommendations can be found in the Supplementary Materials (Table S1).

### 3.2. Hypertensive Heart Disease

For hypertensive heart disease, 10 out of 76 guidelines mentioned vaccine recommendations. These guidelines were from Canada, United States, Europe, United Kingdom and Australia–New Zealand. All ten guidelines recommend the influenza vaccine. Eight out of the ten guidelines recommended the pneumococcal vaccine. One guideline recommended the COVID-19 vaccine.

This paragraph focuses on guidelines recommending the influenza vaccine. In terms of their strength of recommendation, two guidelines mentioned that it was recommended, while eight did not state the strength of recommendation. Additionally, specific indications from the ten guidelines include patients at high risk of developing heart failure and patients with heart failure. The vaccine is recommended for administration annually. Overall, specific contraindications and harms, vaccine administration, and storage details were not mentioned. 

This paragraph focuses on guidelines recommending the pneumococcal vaccine. In terms of their strength of recommendation, two guidelines mentioned that it was recommended, while six did not state the strength of recommendation. Additionally, specific indications include patients at high risk of developing heart failure and patients with heart failure. Overall, specific contraindications and harms, vaccine administration, and storage details were not mentioned.

The strength of recommendation of the COVID-19 vaccine was not stated. It is indicated for patients with heart failure. Specific contraindications and harms, vaccine administration, and storage details were not mentioned. A summary of the above vaccine recommendations can be found in the Supplementary Materials (Table S2).

### 3.3. Atrial Fibrillation and Flutter

For atrial fibrillation and flutter, 1 out of 72 guidelines mentioned vaccine recommendations. That one guideline, from the United States, recommended the influenza and pneumococcal vaccines. The strength of recommendation of both is not stated. They are both indicated for patients with valvular heart disease. Overall, specific contraindications and harms, vaccine administration, and storage details were not mentioned. A summary of the above vaccine recommendations can be found in the Supplementary Materials (Table S3).

### 3.4. COPD

For COPD, 7 out of 39 guidelines mentioned vaccine recommendations. These guidelines were international, and from the United States, Canada, United Kingdom and Australia–New Zealand. All seven guidelines recommended the influenza vaccine. Only six out of seven guidelines recommended the pneumococcal vaccine. Only one guideline, from the Global Initiative for Chronic Obstructive Lung Disease (GOLD), recommended three additional vaccines: coronavirus disease 2019 (COVID-19); the tetanus, diphtheria, pertussis vaccine (Tdap); and zoster, alongside the two aforementioned vaccines.

This paragraph focuses on guidelines recommending the influenza vaccine. In terms of their strength of recommendation, two guidelines were strong, one weak, and four did not state the strength of recommendation. The specific indication for the influenza vaccine is for all patients with COPD. The vaccine is recommended for administration annually. Overall, specific contraindications and harms, vaccine administration, and storage details were not mentioned. 

This paragraph focuses on guidelines recommending the pneumococcal vaccine. In terms of their strength of recommendation, two guidelines were weak, while four did not state this factor. The specific indications include COPD patients below 65 years old, and patients 65 years old and older; adults with COPD, especially those with specific comorbidities or undergoing certain treatments (e.g., chemotherapy); and varying recommendations, depending on smoking and vaccination history. The vaccine is recommended for administration at 50, 65 or at diagnosis of COPD, depending on the patient’s smoking and vaccination history. This is followed by a second, subsequent revaccination. Overall, specific contraindications and harms, vaccine administration, and storage details were not mentioned. 

For COVID-19, the tetanus, diphtheria, pertussis vaccine (Tdap), and zoster vaccines, the strength of recommendation was not stated in the guidelines. The specific indications include all patients with COPD, for the COVID-19 vaccine; those who were not vaccinated in adolescence, for the Tdap vaccine; and adults with COPD ≥ 50 years old, for the Zoster vaccine. For COVID-19, in terms of the timing of the vaccine, the guideline recommended following national guidelines. Overall, specific contraindications and harms, timing for the other two vaccines, vaccine administration, and storage details were not mentioned. A summary of the above vaccine recommendations can be found in the Supplementary Materials (Table S4).

### 3.5. Asthma

For asthma, 3 out of 45 guidelines mentioned vaccine recommendations. These guidelines were international, and from the United States and Australia–New Zealand. All three guidelines recommended the Influenza vaccine. Two out of three guidelines recommended the pneumococcal vaccine. Collectively, one other vaccine (the COVID 19 vaccine) was recommended, alongside the aforementioned vaccines.

Strength of recommendation for all vaccines was not stated in any of the three guidelines. Specific indications include patients with asthma; patients with severe asthma, defined as those who need frequent hospital visits and multiple medicines for asthma; all adults 65 years or more; patients with COPD; pregnant women; and any adult who wishes to avoid influenza. Contraindications to the influenza and pneumococcal vaccines are patients who are receiving high-dose oral steroid therapy. The influenza vaccine is recommended for administration annually. 

Special considerations include that the influenza vaccine should not be given with the expectation that it will reduce either the frequency or severity of asthma exacerbations during the influenza season; that for patients who have documented histories of anaphylactic reactions after ingestion of egg protein and documented evidence of current allergic sensitization to eggs (skin testing or in vitro antigen-specific IgE antibody testing), the risk/benefit ratio of administering of influenza vaccine should be reviewed carefully; and that the first dose of biologic therapy and COVID-19 vaccine should not be given on the same day, to allow the adverse effects of either to be more easily distinguished. Overall, other specific contraindications and harms, vaccine administration, and storage details were not mentioned. A summary of the above vaccine recommendations can be found in the Supplementary Materials (Table S5).

### 3.6. Cirrhosis and Other Chronic Liver Diseases

For cirrhosis and other chronic liver diseases, 3 out of 45 guidelines mentioned vaccine recommendations. These guidelines were from the United States. All three guidelines recommended the hepatitis A and hepatitis B vaccines. Two out of three of the guidelines recommended the pneumococcal and influenza vaccines. Collectively, six other vaccines—Tdap, zoster, HPV, MMR, varicella, and COVID-19 vaccines—were recommended, alongside the previously mentioned vaccines.

Strength of recommendation for all vaccines was not stated in any of the three guidelines. Specific indications include patients with chronic liver disease and patients with alcoholic cirrhosis. Overall, specific contraindications and harms, vaccine administration, and storage details were not mentioned for all vaccines. A summary of the above vaccine recommendations can be found in the Supplementary Materials (Table S6).

### 3.7. Colon and Rectal Cancer

For colon and rectal cancer, 1 out of 46 guidelines mentioned vaccine recommendations. That 1 guideline, from India, recommended the influenza, hepatitis B, MMR, BCG and yellow fever vaccines. The strength of recommendation for all the vaccines was not stated. They are indicated for patients with colorectal cancer undergoing chemotherapy. Some contraindications include that the MMR, BCG and yellow fever vaccines should never be administered to immunocompromised patients, including those receiving chemotherapy, within 6 months of receiving chemotherapy. In terms of timing, the influenza vaccine should be given before chemotherapy, and the hepatitis B vaccine should be given at the end of the chemotherapy cycle. Overall, immunization should be postponed if a patient is suffering from an acute illness. Other vaccine administration information and storage details were not mentioned. A summary of the above vaccine recommendations can be found in the Supplementary Materials (Table S7).

### 3.8. Stomach Cancer

For stomach cancer, 2 out of 22 guidelines mentioned vaccine recommendations. These guidelines were from Canada and India. Both guidelines recommended the influenza, pneumococcal and haemophilus influenza type B (Hib) vaccine. Collectively, four other vaccines—Tdap, polio, varicella zoster, and meningococcal vaccines—were also recommended, on top of the previously mentioned vaccines.

This paragraph focuses on guidelines recommending the influenza, pneumococcal and haemophilus influenza type B (Hib) vaccine. The strength of recommendation was not stated. Additionally, specific indications include patients above 2 years old and patients with gastric lymphoma. The vaccine is recommended to be given 2 to 3 weeks before operation or initiation of anti-lymphoid cancer treatment, and given again 5 years later. Overall, specific contraindications and harms, vaccine administration, and storage details were not mentioned. 

For the Tdap, polio, varicella zoster and meningococcal vaccines, the strength of recommendation was not stated. Specific indications include patients above 2 years old and patients with gastric lymphoma. It is recommended that vaccination is carried out once for the meningococcal vaccine and every 10 years for the Tdap and polio vaccines. The oral polio vaccine is contraindicated in patients with lymphoid cancer. Overall, other specific contraindications and harms, vaccine administration, and storage details were not mentioned. A summary of the above vaccine recommendations can be found in the Supplementary Materials (Table S8).

### 3.9. Diabetes

For diabetes, 7 out of 108 guidelines mentioned vaccine recommendations. These guidelines were international, and from Canada, the United States, United Kingdom, India, Australia–New Zealand and Japan. All seven guidelines recommended the influenza vaccine. Only six out of seven guidelines recommended the pneumococcal vaccine. Collectively, seven other vaccines—Tdap, hepatitis A, hepatitis B, herpes zoster, varicella, human papillomavirus (HPV), and measles, mumps, rubella (MMR)—were also recommended, alongside the two previously mentioned vaccines.

This paragraph focuses on guidelines recommending the influenza vaccine. In terms of their strength of recommendation, one guideline was very strong, one weak and five not stated. Additionally, specific indications from the seven guidelines include all older people with diabetes, and persons with diabetes who are 6 months old and older. One guideline indicated a contraindication of egg allergy, a recent history of Guillain–Barre syndrome within six weeks of a previous influenza vaccination, and febrile illness or any acute infection. The vaccine is recommended for administration annually. Overall, specific contraindications and harms, vaccine administration, and storage details were not mentioned. 

This paragraph focuses on guidelines recommending the pneumococcal vaccine. In terms of their strength of recommendation, one guideline was weak, while five were not stated. Additionally, specific indications from the six guidelines include persons with diabetes aged 19 to 64 years, and people with diabetes 65 years and older or with an immunocompromising condition (e.g., end-stage renal disease). One guideline indicated a contraindication of hypersensitivity to the active substances, or to any of the excipients of the vaccine febrile illness, or any acute infection. The vaccine is recommended for administration at the time of diagnosis, with a second and third dose later on in life. Overall, specific contraindications and harms, vaccine administration, and storage details were not mentioned. 

For the seven other vaccines—Tdap, hepatitis A, hepatitis B, herpes zoster, varicella, human papillomavirus (HPV), and measles, mumps, rubella (MMR)—the strength of recommendation for the guidelines varied from not stated to very strong. Specific indications include all unvaccinated patients with diabetes, for the hepatitis B vaccine; females and diabetic patients, for the HPV vaccine; and T2DM patients aged 70 to 79 years old, for the zoster vaccine. In terms of the timing of the vaccinations, the guidelines recommend that the hepatitis B vaccine be given at the diagnosis of diabetes; that Tdap be given every 10 years, following the completion of the primary series in routine childhood vaccination; that one or two doses of MMR vaccine be given 4 weeks apart; that two doses of Varicella vaccine be given 4 weeks apart; that the zoster vaccine be given once at 60 years old; that two doses of the hepatitis A vaccine be given 6 months apart; and that three doses of the HPV vaccine be given, up to the age of 26. Overall, specific contraindications and harms, vaccine administration, and storage details were not mentioned. A summary of the above vaccine recommendations can be found in the Supplementary Materials (Table S9).

### 3.10. CKD

For chronic kidney disease, 6 out of 46 guidelines mentioned vaccine recommendations. These guidelines were international, and from the United States, Australia–New Zealand and Japan. Five out of six of the guidelines recommended both the pneumococcal and influenza vaccine. Four out of six of the guidelines recommended the hepatitis B vaccine. Collectively, six other vaccines—Tdap, MMR, varicella, zoster/shingles, varicella, hepatitis A—were also recommended alongside the three previously mentioned vaccines.

This paragraph focuses on guidelines recommending the influenza vaccine. Their strength of recommendation ranged from a grade 1 strength of recommendation to a grade 2 level of recommendation (i.e., strong); some guidelines did not state a strength of recommendation. Additionally, specific indications from the six guidelines include adults with CKD and/or diabetes. Specific contraindications mentioned include giving a live attenuated influenza vaccine to CKD patients. The vaccine is recommended for administration annually. Overall, storage details were not mentioned. 

This paragraph focuses on guidelines recommending the pneumococcal vaccine. Their strength of recommendation ranged from a grade 1 strength of recommendation to a grade 2 level of recommendation (i.e., strong); some guidelines did not state a strength of recommendation. Additionally, specific indications include adults aged ≥ 19 years with immunocompromising conditions (including those with chronic renal failure or nephrotic syndrome), functional or anatomic asplenia, cerebrospinal fluid (CSF) leaks, or cochlear implants; eGFR < 30 mL/min/1.73 m^2^ (GFR categories G4–G5), and those at high risk of pneumococcal infection (e.g., nephrotic syndrome, diabetes, or those receiving immunosuppression); all adults aged 65 years and older; and adults at high risk aged 19 to 64 years. Revaccination is recommended within 5 years. Overall, specific contraindications and harms, vaccine administration, and storage details were not mentioned. 

For the six other vaccines (Tdap, MMR, varicella, zoster/shingles, varicella, and hepatitis A), the strength of recommendation for the guidelines varied from a grade 1 strength of recommendation to strong. Specific indications include all susceptible chronic haemodialysis patients; pre-end-stage renal disease patients before they become dialysis dependent; and a history of HCV infection (whether NAT-positive or not). The hepatitis B vaccine requires booster doses, with a four-dose schedule (20 ug [1.0 mL doses]) administered in one or two injections. Overall, for the rest of the vaccines, specific contraindications and harms, vaccine administration, and storage details were not mentioned. A summary of the above vaccine recommendations can be found in the Supplementary Materials (Table S10).

### 3.11. Stroke

For stroke, 3 out of 62 guidelines mentioned vaccine recommendations. These guidelines were from the United States and Canada. All three guidelines recommended the influenza vaccine. The guidelines from the United States recommended that the vaccinations be taken annually.

In terms of their strength of recommendation, one guideline had level B evidence from randomized controlled trials, one was moderate, and one did not state a strength of recommendation. Only one guideline mentioned specific indications, which was in patients with pre-existing cardiovascular risk factors. Overall, specific contraindications and harms, vaccine administration, and storage details were not mentioned. A summary of the above vaccine recommendations can be found in the Supplementary Materials (Table S11).

## 4. Discussion

Detailed information about the vaccinations, including contraindications, route of administration, dosage, and storage, is mostly missing in the guidelines to be discussed in this section. This could be because many countries have national public health agencies that consolidate this information. These include the Centers for Disease Control and Prevention in the United States [16], and the Department of Health and Aged Care in Australia [17].

However, it should be noted that there is more information regarding the recommended influenza vaccine. This could be due to the fact that influenza vaccines are well established in terms of their development, mechanism of action, ingredients, and contraindications [18].

### 4.1. Discussion for Each Recommended Vaccine

#### 4.1.1. Influenza Vaccine

In general, individuals with pre-existing diseases are at much higher risk of complications of influenza infections [19]. Influenza vaccines have been found to effectively decrease mortality due to influenza infections among elderly patients [20].

The following is a discussion about the rationale behind recommending the influenza vaccination to patients with the respective diseases.

##### Cardiac-Related Diseases

Influenza vaccination has evidently lowered the risk of cardiac and non-cardiac mortality in elderly patients with ischaemic heart disease [21,22]. The influenza vaccine also has evidently reduced the overall morbidity and mortality of diabetic patients and those with hypertensive heart disease [21,23,24].

Patients with underlying atrial fibrillation have a worse prognosis with influenza infection [25]. Influenza infection also increases the risk of haemorrhagic [26] and ischaemic stroke [27] in patients with atrial fibrillation. Therefore, the influenza vaccination is recommended for its protective effects.

##### Respiratory-Related Diseases

Influenza infection is known to be associated with COPD exacerbations, stroke, respiratory failure and pneumonia in patients with COPD [28]. Influenza vaccination can effectively reduce these events in COPD patients [29]. Similarly, in patients with asthma, influenza vaccination effectively lowers the rate of asthma exacerbations requiring Accident and Emergency department visits and/or hospitalisations, and influenza infections [30,31].

##### Gastrointestinal-Related Diseases

The efficacy of the influenza vaccine in patients with chronic liver disease is uncertain, due to the lack of research studies with a substantial sample size [32,33]. However, since influenza infection is known to cause the decompensation of liver cirrhosis and to increase mortality [34], many still recommend the influenza vaccination for patients with liver cirrhosis [35].

Cancer patients, such as those with colorectal and stomach cancer, especially those undergoing or planning to undergo chemotherapy, are recommended for vaccination against influenza. This is because influenza infection has been observed to delay chemotherapy [36], and results in severe complications in these immunocompromised patients [37]. As such, the influenza vaccine has been associated with lower mortality in cancer patients [38].

##### Other Diseases

In diabetic patients, influenza vaccination has been proven to reduce the overall morbidity and mortality related to influenza infection and cardiovascular events [39,40].

The influenza vaccine has been found to lower the morbidity of CKD patients from coronary heart disease [41], heart failure [42], and dementia [43], and even decreases the incidence of lung cancer in patients with CKD [44].

Studies have also revealed that influenza vaccination reduces the risk of all types of strokes, to a varying extent, regardless of the baseline risk of stroke [45], as influenza infection has been suggested to precipitate stroke [46].

##### Vaccine Administration Details

Most guidelines did not specify which type of influenza vaccine is recommended. For diabetes patients, specific types of influenza vaccines were recommended.

In guidelines written for diabetes, two guidelines specifically recommended the quadrivalent influenza vaccine, which has been available in the developed nations since 2012. Quadrivalent vaccines include an additional strain of influenza B virus compared to the trivalent vaccines. It has been estimated that the quadrivalent influenza vaccine has higher protective effects and higher cost-effectiveness than trivalent influenza vaccines [47,48].

Some guidelines for hypertensive heart disease and stomach cancer have specifically recommended that the elderly be vaccinated in fall, because studies have shown that the incidence of influenza infection peaks in winter [49].

For patients with colorectal cancer, it is recommended that influenza vaccination be offered before chemotherapy for optimal protection during chemotherapy treatment, as this treatment causes the patient to be in an immunocompromised state [50].

#### 4.1.2. Pneumococcal Vaccine

Those with pre-existing medical conditions are immunocompromised and are at a higher risk of contracting pneumococcal pneumonia and having complications. Pneumococcus is also the most common cause of community-acquired pneumonia, demonstrating high incidence rates of invasive pneumococcal disease among adults above the age of 65 [51].

##### Cardiac-Related Diseases

In patients with cardiovascular disease with pneumococcal infections, the severity and risk of complications is higher. Hypertensive heart disease and atrial fibrillation, two of the cardiac conditions analyzed in this paper, are associated with a higher risk of stroke, cardiovascular disease (such as ischaemic heart disease), and even cardiovascular death [52,53]. Pneumococcal vaccinations have cardioprotective effects, reducing the risk of myocardial infarctions and decreasing morbidity and mortality.

##### Respiratory-Related Diseases

COPD patients are more susceptible to respiratory infections due to the impaired mucociliary clearance mechanisms and increased mucus production, which allow for increased bacterial and viral attachment. Use of pneumococcal vaccinations in COPD patients prevents exacerbations from respiratory tract infections, and is therefore recommended [54]. Guidelines for asthma were also analyzed in this study. Respiratory infections, especially community-acquired pneumonia, are one of the main causes of asthma exacerbations [55]. Use of the pneumococcal vaccination among asthma patients has been documented to have prompted a decrease in pneumococcal pneumonia-related hospitalizations [56].

##### Gastrointestinal-Related Diseases

Patients with severe liver disease have increased mortality and morbidity due to S. pneumoniae infections. In a study conducted among 45 unimmunised patients with end-stage liver disease who were vaccinated during liver transplantation evaluation, a significant response to the 23-valent pneumococcal vaccine was observed [57]. As such, the pneumococcal vaccine plays a critical role in the management of patients with chronic liver disease.

The pneumococcal vaccine is also recommended for patients with stomach cancer. Cancer patients are at higher risk of invasive pneumococcal disease (IPD) compared to the general population, with immunocompromised cancer patients contributing to 17–37% of all IPD cases [58].

The vaccine was also not recommended in the colorectal cancer guidelines. A study conducted in Taiwan identified 120,605 elderly patients with colorectal cancer, and explored the effectiveness of the pneumococcal vaccine in these patients. It found that the PPSV23 vaccine significantly reduced the rate of pneumonia hospitalization in elderly patients, and that the pneumonia-free survival rate was significantly higher in vaccinated patients compared to unvaccinated ones [59]. The lack of pneumococcal vaccine recommendations in the guidelines could be due to the fact that only one guideline even mentioned vaccines. A larger database of guidelines might yield better results. 

##### Other Diseases

Patients with DM are at increased risk of acquiring pneumonia and invasive pneumococcal disease. They are also six times more likely to be hospitalized, and three times more likely to die from complications of influenza or pneumonia, than those in the general population [60]. The use of pneumococcal vaccinations significantly lowers the risk of morbidity and mortality.

The use of PPV-23 and PCV-13 is recommended in CKD patients. CKD patients have decreased B and CD4+ lymphocytes, and are at high risk of infections. Streptococcus pneumoniae is one of the main causes of community-acquired pneumonia in dialysis and kidney transplant patients [61].

The pneumococcal vaccine was not mentioned in any of the stroke guidelines. This could be due to the fact that many clinical studies report the vaccine having no effect on stroke risk. A study conducted by Kaiser Permanente concluded that the pneumococcal vaccine was not associated with reduced stroke risk. There were 5.30 stroke events per 1000 vaccinated person years, and 1.90 per 1000 unvaccinated person years [62]. These results were corroborated by another study based on data from the United Kingdom. The pneumococcal vaccine was found to have no significant effect on stroke or transient ischaemic attack risk [63]. 

##### Vaccine Administration Details

Out of the 33 guidelines from different countries, among five diseases, only 9 guidelines provided information about the type of pneumococcal vaccine recommended. Five guidelines recommended both the PPV-23 and PCV-13 vaccines. Five guidelines recommended only the PPV-23 vaccine. One guideline from the American Association of Clinical Endocrinology recommended the PCV 15 and 20 vaccines as well.

In a study conducted among outpatients aged ≥ 65 years with chronic respiratory diseases in Shizuoka General Hospital, Japan, PPSV-23 was proven to be effective in preventing pneumococcal pneumonia among the elderly. Out of 320 patients vaccinated with PPSV-23, 1.88% developed pneumococcal pneumonia compared to 4.05% of the 3898 unvaccinated patients [64]. The effectiveness of the PPSV-23 vaccine explains why the majority of guidelines recommend the PPV-23 vaccine.

The combined use of PPSV-23 and PCV-13 is also highly recommended by several guidelines. A 2013 study involving 936 adults aged 70 years and older highlighted the limitations of using PPSV23 alone, and suggested that it might be more effective to administer PCV13 following initial vaccination with PPSV-23 [65]. The combination of PPSV-23 with PCV-13 has been documented to produce a superior immune response to PPSV-23 alone, which would allow for an overall reduction in the severity of pneumococcal pneumonia.

There are specific indications stated for chronic kidney disease. The pneumococcal vaccine is recommended for patients with eGFR < 30 mL/min/1.73 m^2^ (GFR categories G4–G5) and those at high risk of pneumococcal infection (e.g., nephrotic syndrome, diabetes, or those receiving immunosuppression). As CKD progresses, hospitalization rates and the risk of pneumococcal infection increases. Therefore, patients with more severe CKD must be protected against pneumococcal pneumonia [66].

As for the timing of the vaccine, the PPSV-23 vaccine is recommended for administration at time of diagnosis, and a single revaccination within 5 years. The second dose of PPSV-23 is recommended to be at least 5 years apart from the first dose. If the PSV-13 vaccine is co-administered, it should be given first and followed at least 8 weeks later by the PPSV-23 vaccine. The recommended interval between the PPSV-23 and PCV-13 vaccine is extended up to 1 year or even 5 years in some guidelines. If the PCV-15 vaccine is used, PPSV-23 should be administered at least 12 months after.

For stomach cancer, there are specific considerations involved in the timing of the pneumococcal vaccine. It is recommended for administration at least 2 weeks prior to initiation of anti-lymphoid cancer treatment or splenectomy, and to be repeated 5 years later (this applies to both vaccines). However, according to a study conducted in three cancer centres in Korea from March 2016 to March 2018, administering the vaccine on day 1 of treatment in patients with gastric cancer is not inferior to administering the vaccine 2 weeks prior [67]. The paper attributed the known timing of vaccine administration 2 weeks prior to treatment to the lack of studies exploring the optimal timing required.

#### 4.1.3. Hepatitis Vaccine

##### Gastrointestinal-Related Diseases

A possible reason that only four diseases had a recommendation for hepatitis vaccines could be the increased susceptibility to hepatitis infections of patients who have these diseases and are immunocompromised. Diabetes has emerged as a risk factor for increased complications in patients with acute viral hepatitis [68]. The hepatitis B virus is one of the major causes of chronic liver disease, and it can cause many extrahepatic complications and manifestations, including renal failure and various nephropathies [69]. However, it has been found that vaccination of haemodialysis patients with a combined hepatitis A and hepatitis B vaccine results in increased seroprotection against the hepatitis B virus, compared to the hepatitis B monovalent vaccine [70]. This explains the recommendation that both the hepatitis A and B vaccine be given together (instead of a hepatitis B monovalent vaccine) in patients with chronic kidney disease.

Acute hepatitis A and B against the the background of chronic liver disease are associated with more severe liver disease and a higher fatality rate, thus explaining why hepatitis A and B vaccinations are recommended for these patients [71]. A possible explanation for India being the only country to recommend hepatitis vaccines for colon and rectal cancer is that viral hepatitis is a major health challenge in India, and therefore the hepatitis vaccine is recommended for various diseases, and not just specifically colon and rectal cancer [72]. This can be observed in a guideline from India that also recommends the hepatitis A vaccine for patients with diabetes.

##### Cardiac-Related Diseases 

Conversely, there are possible reasons as to why the other seven non-communicable diseases did not recommend the hepatitis vaccine. There is research that suggests that hepatitis vaccines do not have an impact on decreasing the risk of ischaemic heart disease [73]. Additionally, there is also a report that mentions HBV infection being associated with a lower risk of developing stroke; however, further research is required to confirm this [74]. 

##### Respiratory-Related Diseases and Other Diseases

For respiratory-related diseases and other diseases, at the time of writing, there are no papers that provide strong evidence for contraindication to hepatitis vaccines or any possible side effects in the elderly population. 

##### Vaccine Administration Details

In terms of the details of hepatitis vaccine recommendations, all the guidelines for cirrhosis and other chronic liver diseases do not state any strength of recommendation or any details of vaccination, such as specific contraindication, harm, timing, route, dosage, and storage. For the other three diseases, there is some information provided, but these details are largely missing.

#### 4.1.4. COVID-19 Vaccine

COVID-19 vaccination is recommended by some guidelines concerning COPD, diabetes, chronic liver disease and asthma, published in 2022. This is because patients with COPD, asthma, diabetes, and liver cirrhosis are at higher risk of severe COVID-19 infection [75,76,77].

#### 4.1.5. Varicella Zoster Vaccine

Studies have shown that patients with COPD, diabetes, chronic kidney disease, chronic liver disease, and stomach cancer are at higher risk of zoster infection [78]. This is particularly pertinent for patients with diabetes and/or renal diseases, as they have a 1.8- to 8.4-fold higher risk of zoster infections than patients with other underlying diseases [79]. There is a much lower risk of zoster reactivation, relative to other infections such as pneumococcus and hepatitis infections, in patients with chronic liver disease [80]. Varicella zoster vaccination is recommended for gastric lymphoma patients. Although no reasoning can be found specifically for gastric lymphoma patients, varicella zoster vaccines have been found to be immunogenic in patients with solid organ tumours, with no significant safety concerns [81].

#### 4.1.6. Tdap Vaccine

Although we vaccinate children with Tdap vaccines, the seroprevalence of diphtheria, tetanus, and pertussis is low in the elderly [82]. Tdap vaccines and their boosters are recommended in the guidelines written for COPD, diabetes, chronic kidney disease, chronic liver disease, and stomach cancer. A relatively significant percentage of elderly people with severe pertussis infections in United States from 2011–2015 were found to have underlying diseases such as diabetes and renal dysfunction, suggesting a correlation between these diseases and the development of severe pertussis infection [83]. COPD and asthma also evidently increase the risk of severe pertussis infection; pertussis infection, similarly, exacerbates asthma and COPD [84]. This explains why Tdap vaccines are recommended for patients with these diseases; surprisingly, Tdap vaccination is not recommended in the guidelines written for asthma. There is limited knowledge on the efficacy of Tdap in patients with chronic liver disease and/or cirrhosis [32]. On the other hand, poor efficacy in elderly has been reported for vaccinations against diphtheria and tetanus [85,86,87]. Limited studies have evaluated the efficacy of tetanus vaccine in protecting patients with stomach cancers from Clostridium tetani, and instead, more studies are exploring the therapeutic effects of tetanus toxoid in treating stomach cancers [88].

#### 4.1.7. MMR and Varicella Vaccine

There are mixed opinions regarding re-vaccinating elderly people with these childhood vaccines for protection against vaccine-preventable diseases. Some believe that since the immunity of the elderly has waned over the years, re-vaccinating therefore protects them, but there is a lack of evidence for this [89]. With the recent resurgence of measles infections, there is the potential of the benefits outweighing the risks of re-vaccinating the elderly [90].

The MMR vaccine is also recommended for patients with colorectal cancer who are no longer immunocompromised. It has been found that the seroprevalence of measles and mumps antibodies is low among cancer patients, meaning their risk of measles and mumps infection is increased during community outbreaks [91].

#### 4.1.8. HPV Vaccine

It has been found that patients with diabetes have more extensive infections and higher chances of the recurrence of genital warts, which are caused by HPV [92]. The HPV vaccine has been shown to be effective in reducing the incidence of genital warts secondary to HPV [93]. Limited studies have been carried out, showing mixed results concerning the efficacy of the HPV vaccine in patients with chronic liver disease [94].

#### 4.1.9. Meningococcal Vaccine

Gastric lymphoma patients that are undergoing or have undergone splenectomy are at a higher risk of infection with encapsulated organisms, and as such, they should be vaccinated against these bacteria every five years [95].

#### 4.1.10. Poliovirus Vaccine

It has been found that unless the patients with gastric lymphoma have a low antibody titre or have undergone hematopoietic stem cell transplantation, revaccination with the polio vaccine is not absolutely necessary [96].

#### 4.1.11. Yellow Fever Vaccine

Yellow fever vaccination is recommended by one of the guidelines written for colon and rectal cancer. However, no clear evidence has been found to substantiate this recommendation.

### 4.2. Strengths and Limitations

This study has several strengths, including disease identification and focusing on the age group of 75 and above.

The diseases to be referenced were selected from the Global Burden of Disease Study 2019. This study collects data about “premature death and disability from more than 350 diseases and injuries in 195 countries”. The large sample size and its extensiveness across different countries makes this study more reliable and representative of the world population. Composite indicators such as incidence, prevalence, mortality, years of life lost (YLLs), years lived with disability (YLDs), and disability-adjusted life years (DALYs) are used to assess disease burden. These allow for quantifiable measures to compare the burden of various diseases.

The focus on the older age group is a great strength of this study. Vaccination guidelines vary across different age groups, especially between children and adults. Each country follows its childhood immunization schedule based on the WHO position paper on routine immunizations for children. The vaccination guideline recommendations for children are more established and adhered to than those for adults. With decreasing vaccination compliance and increasing prevalence of diseases causing mortality and morbidity among older persons, narrowing the focus to older persons allows for this study to be more valuable in advising healthcare policies and practices.

One limitation of this study is the use of UpToDate to identify the guidelines to be examined. Although UpToDate is a resource accredited and recognized by experts and institutions around the world, there might be selection bias in the information and guidelines chosen to be listed on it. Only guidelines from selected countries, including the United States, Europe, Canada, United Kingdom, Australia–New Zealand, Japan, etc., were listed. This is not an accurate representation of the global population, and the compiled vaccination guidelines may not be applicable to every country.

Another limitation is the exclusion of non-English guidelines due to language issues. Many papers from Asian countries such as Japan were in the authors’ native language, and the information in the guidelines could therefore not be examined. This resulted in guidelines applicable to the Asian population being less represented in this study.

### 4.3. Clinical Implications and Future Directions

The current guidelines for older adults do not commonly suggest vaccination for certain conditions. For those that do, the recommendations often lack information on potential negative effects and the proper administration of the vaccine.

This may be because there is not enough evidence to support the guidelines; the guidelines may also need to include vaccination guidance more frequently, and provide more specific information.

A possible strategy to combat this is to collaborate with international organizations, such as the World Health Organization (WHO) or the Centers for Disease Control and Prevention (CDC), to share information, insights, and best practices. International collaboration can help fill information gaps and ensure standardisation in vaccine guidelines worldwide. Additionally, more time and resources should be allocated to conducting thorough scientific research on vaccines. This will involve working with reputable sources, such as scientific journals, public health organizations, and regulatory bodies, to gather as much information as possible about a given vaccine’s potential negative effects and proper administration.

As a result, our work aims to encourage further research into vaccination for older adults, and to push for guidelines to provide more comprehensive recommendations. However, it is important to study whether these guideline improvements would ultimately lead to better clinical outcomes.

## 5. Conclusions

To conclude, vaccines still remain the most cost-effective health intervention, especially amongst vulnerable populations such as the elderly. Through our thorough review of guidelines for vaccination in adults 75 years old and above, we have identified many gaps that could potentially be filled and looked into by our global health system. Filling said gaps may potentially improve elderly people’s quality of life, and reduce their disability-adjusted life years. Future studies should now follow up and review these guidelines, as more guidance is being put in place, including non-English guidelines.

## Data Availability

The data used in this research paper were obtained from Uptodate, an online medical resource. Access to Uptodate’s database and content is subject to their terms of use and subscription agreement. Researchers interested in accessing the data used in this study should refer to Uptodate’s website (www.uptodate.com) for information on how to obtain access to their platform.

## Supplementary Materials

The following supporting information can be downloaded at: https://www.mdpi.com/article/10.3390/vaccines11061076/s1, Table S1: Summary of vaccination recommendation for IHD; Table S2: Summary of vaccination recommendation for Hypertensive heart disease; Table S3: Summary of vaccination recommendation for Atrial Fibrillation and Flutter; Table S4: Summary of vaccination recommendation for COPD; Table S5: Summary of vaccination recommendation for Asthma; Table S6: Summary of vaccination recommendation for Cirrhosis and Other Chronic Liver Diseases; Table S7: Summary of vaccination recommendation for Colon and rectum cancer; Table S8: Summary of vaccination recommendation for Stomach Cancer; Table S9: Summary of vaccination recommendation for Diabetes; Table S10: Summary of vaccination recommendation for CKD; Table S11: Summary of vaccination recommendation for Stroke.
