# Supplementary material for: Review of Vaccination Recommendations in Guidelines for Non-Communicable Diseases with Highest Global Disease Burden among Adults 75 Years Old and Above
Source: Vaccines (Basel). 2023 Jun 8;11(6):1076. doi: 10.3390/vaccines11061076 (PMC10305037; doi:10.3390/vaccines11061076)
Supplement: Supplementary file 1 [file vaccines-11-01076-s001.zip › vaccines-2371220-supplementary.pdf]

Supplementary Materials

Cardiac-related diseases

Table S1. Summary of vaccination recommendation for IHD.

Name of guidelines (Year of guideline publication), Country of origin of the guideline	Name of recommended vaccines, Strength of recommendation (strong/moderate/weak/not stated)	Details of recommendation for each vaccine		
		Specific indications (age, severity of disease, disease subgroup)	Specific contraindications and harms (side effects)	Vaccine administration (timing, route, dosage) and storage details
ACCF/AHA/American College of Physicians (ACP)/AATS/ PCNA/SCAI/STS: Guideline for the diagnosis and management of patients with stable ischemic heart disease (2012), United States	Inactivated Influenza vaccine, Strength of recommendation: Strong	Stable Ischemic Heart Disease patients ≥65 years old	Contraindications: Not mentioned Harms: Pain and myalgia at injection site	Timing: Annual Route: Intramuscular Dosage: Standard dose Storage: Not mentioned
AHA: A scientific statement for secondary prevention after coronary artery bypass graft (2015), United States	Influenza vaccine, Strength of recommendation: Strong	All CABG patients, unless contraindications exist	Not mentioned	Timing: Uncertain Route: Not mentioned Dosage: Not mentioned Storage: Not mentioned
AHA: Scientific statement on self-care for the prevention and management of cardiovascular disease and stroke (2017), United States	Influenza vaccine, Strength of recommendation: Not stated	Not mentioned	Not mentioned	Timing: Annual Route: Not mentioned Dosage: Not mentioned Storage: Not mentioned
European Society of Cardiology (ESC): Guidelines on chronic coronary syndromes (2019), Europe	Influenza vaccine, Strength of recommendation: Strong	Chronic Coronary Syndrome patients especially in the elderly (age 65 and above)	Not mentioned	Timing: Annual Route: Not mentioned Dosage: Not mentioned Storage: Not mentioned
European Society of Cardiology (ESC): Guidelines on cardiovascular disease prevention in clinical practice (2021), Europe	Influenza vaccine, Strength of recommendation: Not stated	Not mentioned	Not mentioned	Not mentioned

NHFA and CSANZ: Reducing risk in heart disease – An expert guide to clinical practice for secondary prevention of coronary heart disease (2012), Australia-New Zealand	Influenza vaccine, Strength of recommendation: Not stated	Everyone with Coronary Heart Disease unless contraindicated	Not mentioned	Timing: Annual Route: Not mentioned Dosage: Not mentioned Storage: Not mentioned
	Pneumococcal vaccine, Strength of recommendation: Not stated	Everyone with Coronary Heart Disease unless contraindicated	Not mentioned	Not mentioned

Table S2. Summary of vaccination recommendation for Hypertensive heart disease.

Name of guidelines (Year of guideline publication), Country of origin of the guideline	Name of recommended vaccines, Strength of recommendation (strong/moderate/weak/not stated)	Details of recommendation for each vaccine		
		Specific indications (age, severity of disease, disease subgroup)	Specific contraindications and harms (side effects)	Vaccine administration (timing, route, dosage) and storage details
CCS: Society guidelines for the management of heart failure, comprehensive update (2017), Canada	Influenza vaccine, Strength of recommendation: Not stated	Patients at high risk for developing Heart Failure	Not mentioned	Not mentioned
	Pneumococcal pneumonia vaccine, Strength of recommendation: Not stated		Not mentioned	Not mentioned
AHA: Scientific statement on contributory risk and management of comorbidities of hypertension, obesity, diabetes mellitus, hyperlipidemia, and metabolic syndrome in chronic heart failure (2016), United States	Flu vaccine, Strength of recommendation: Not stated	Heart Failure patients	Not mentioned	Not mentioned
	Pneumonia vaccine, Strength of recommendation: Not stated		Not mentioned	Not mentioned
AHA/HFSA: Scientific statement on heart failure management in skilled nursing facilities (2015), United States	Influenza vaccine, Strength of recommendation: Not stated	Patients with Heart Failure	Not mentioned	Timing: Every fall Route: Not mentioned Dosage: Not mentioned Storage: Not mentioned
	Pneumococcal vaccine polyvalent, Strength of recommendation: Not stated		Not mentioned	Timing: 1 dose at any age and repeat at age ≥65 years if prior dose was given before age 65 years and 5 years

				has elapsed since first dose<br><br>Route: Not mentioned<br>Dosage: Not mentioned<br>Storage: Not mentioned
HFSA: Heart failure guidelines (2010), United States	Influenza vaccine, Strength of recommendation: Recommended (Part of routine care Exceptions to therapy should be minimized)	All patients with Heart Failure with no known contraindications	Not mentioned	Timing: Annual<br>Route: Not mentioned<br>Dosage: Not mentioned<br>Storage: Not mentioned
	Pneumococcal vaccine, Strength of recommendation: Recommended (Part of routine care Exceptions to therapy should be minimized)		Not mentioned	Not mentioned
AHA: Scientific statement on promoting self-care in persons with heart failure (2009), United States	Flu vaccine, Strength of recommendation: Not stated	Patients with Heart Failure	Not mentioned	Timing: Annual<br>Route: Not mentioned<br>Dosage: Not mentioned<br>Storage: Not mentioned
	Pneumococcal vaccine, Strength of recommendation: Not stated	Persons over the age of 65 years unless contraindicated	Not mentioned	Not mentioned
ESC: Guidelines for the diagnosis and treatment of acute and chronic heart failure (2021), Europe	Influenza vaccine, Strength of recommendation: Not stated	Patients with Heart Failure	Not mentioned	Not mentioned
	Pneumococcal vaccine, Strength of recommendation: Not stated		Not mentioned	Not mentioned
	COVID-19 vaccine, Strength of recommendation: Not stated		Not mentioned	Not mentioned
European Society of Cardiology (ESC): Guidelines on diagnosis and management of hypertrophic cardiomyopathy (2014), Europe	Influenza vaccine, Strength of recommendation: Not stated	Symptomatic patients with hypertrophic cardiomyopathy, in the absence of contraindications	Not mentioned	Timing: Annual<br>Route: Not mentioned<br>Dosage: Not mentioned<br>Storage: Not mentioned
NICE: Guideline on chronic heart failure in adults – Diagnosis and	Influenza vaccine, Strength of recommendation: Not stated	Patients with Heart Failure	Not mentioned	Timing: Annual<br>Route: Not mentioned<br>Dosage: Not

management (2018), United Kingdom				mentioned<br>Storage: Not mentioned
Scottish Intercollegiate Guidelines Network (SIGN): Management of chronic heart failure – A national clinical guideline (2016), United Kingdom	Influenza vaccine, Strength of recommendation: Recommended best practice	Patients with Chronic Heart Failure	Not mentioned	Timing: Annual<br>Route: Not mentioned<br>Dosage: Not mentioned<br>Storage: Not mentioned
	Pneumococcal vaccine, Strength of recommendation: Recommended best practice		Not mentioned	Not mentioned
National Heart Foundation of Australia (NHFA) and Cardiac Society for Australia and New Zealand (CSANZ): Guidelines for the prevention, detection, and management of heart failure in Australia (2018), Australia-New Zealand	Influenza vaccine, Strength of recommendation: Not stated	Patients with Heart Failure	Not mentioned	Not mentioned
	Pneumococcal vaccine, Strength of recommendation: Not stated		Not mentioned	Not mentioned

Table S3. Summary of vaccination recommendation for aAtrial Fibrillation and Flutter.

Name of guidelines (Year of guideline publication), Country of origin of the guideline	Name of recommended vaccines, Strength of recommendation (strong/moderate/weak/not stated)	Details of recommendation for each vaccine		
		Specific indications (age, severity of disease, disease subgroup)	Specific contraindications and harms (side effects)	Vaccine administration (timing, route, dosage) and storage details
AHA/ACC/Heart Rhythm Society (HRS): Guideline for the management of patient with atrial fibrillation, focused update (2019), United States	Influenza, Strength of recommendation: Not stated	Patients with VHD	Not mentioned	Not mentioned
	Pneumococcal, Strength of recommendation: Not stated		Not mentioned	Not mentioned

Respiratory- related diseases

Table S4. Summary of vaccination recommendation for COPD.

		Details of recommendation for each vaccine

Name of guidelines (Year of guideline publication), Country of origin of the guideline	Name of recommended vaccines, Strength of recommendation (strong/moderate/weak/not stated)	Specific indications (age, severity of disease, disease subgroup)	Specific contraindications and harms (side effects)	Vaccine administration (timing, route, dosage) and storage details
Global Initiative for Chronic Obstructive Lung Disease (GOLD): Global strategy for the diagnosis, management, and prevention of chronic obstructive pulmonary disease, update (2022), International	Influenza vaccine (either killed or live inactivated vaccines), Strength of recommendation: Not stated	All patients with COPD	Not mentioned	Not mentioned
	SARS-Cov-2 (COVID-19), Strength of recommendation: Not stated	All patients with COPD	Not mentioned	Timing: Follow national guidelines<br>Route: Not mentioned<br>Dosage: Not mentioned<br>Storage: Not mentioned
	23-valent pneumococcal polysaccharide vaccine (PPSV23), Strength of recommendation: Not stated	COPD patients <65 years old, and patients ≥65 years old	Not mentioned	Not mentioned
	13-valent conjugated pneumococcal vaccine (PCV13), Strength of recommendation: Not stated	General population ≥65 years old	Not mentioned	Not mentioned
	Tdap (dTAP/dTPa), Strength of recommendation: Not stated	For those who were not vaccinated in adolescence	Not mentioned	Not mentioned
	Zoster, Strength of recommendation: Not stated	Adults with COPD ≥50 years old	Not mentioned	Not mentioned
BC Guidelines: Chronic obstructive pulmonary disease (COPD) – Diagnosis and management (2017, updated 2020), Canada	Influenza vaccine, Strength of recommendation: Not stated	All patients with COPD	Not mentioned	Timing: Annual<br>Route: Not mentioned<br>Dosage: Not mentioned<br>Storage: Not mentioned
	Pneumococcal polysaccharide vaccine, Strength of recommendation: Not stated	Adults with COPD especially those with specific comorbidities or undergoing certain treatments (e.g., chemotherapy)	Not mentioned	Not mentioned

American College of Chest Physicians (CHEST) and CTS: Guideline for the prevention of acute exacerbations of COPD (2015), Canada and United States	Influenza vaccine, Strength of recommendation: Strong	For all persons aged ≥6 months who do not have contraindications, especially COPD patients	Not mentioned	Timing: Annual Route: Not mentioned Dosage: Not mentioned Storage: Not mentioned
	23-valent pneumococcal vaccine, Strength of recommendation: Weak	All adults aged ≥65 years and in those aged 19-64 years with COPD	Not mentioned	Not mentioned
American Thoracic Society (ATS): An official workshop report on challenges in the implementation of chronic obstructive pulmonary disease guidelines in low- and middle-income countries (2021), United States	Influenza vaccine, Strength of recommendation: Not stated	All patients with COPD	Not mentioned	Not mentioned
Department of Veterans Affairs (VA)/Department of Defense (DoD): Clinical practice guideline for the management of chronic obstructive pulmonary disease (2021), United States	Influenza vaccine, Strength of recommendation: Weak	All patients with COPD	Not mentioned	Not mentioned
	Pneumococcal vaccine, Strength of recommendation: Weak	All patients with COPD	Not mentioned	Not mentioned
NICE: Guideline on chronic obstructive pulmonary disease in over 16s – Diagnosis and management (2018, updated 2019), United Kingdom	Influenza vaccine, Strength of recommendation: Not stated	All patients with COPD	Not mentioned	Timing: Annual Route: Not mentioned Dosage: Not mentioned Storage: Not mentioned
	Pneumococcal vaccine, Strength of recommendation: Not stated	All patients with COPD	Not mentioned	Not mentioned
Lung Foundation Australia (LFA) and Thoracic Society of Australia and New Zealand (TSANZ): The COPD-X plan – Australian and New Zealand guidelines for the management of chronic obstructive pulmonary disease	Influenza vaccine, Strength of recommendation: Strong	All patients with COPD	Not mentioned	Timing: Annual Route: Not mentioned Dosage: Not mentioned Storage: Not mentioned
	Pneumococcal vaccine (23vPPV), Strength of	Not at increased risk of IPD*, / Non-indigenous:	Not mentioned	Timing: -Initial dose: at 65 years old -First

(2021), Australia-New Zealand	recommendation: Not stated	non-smoker			revaccination: Nil<br>-Second revaccination: Nil<br>Route: Not mentioned<br>Dosage: Not mentioned<br>Storage: Not mentioned
			Indigenous:		Timing:<br>-Initial dose: At 50 years old<br>-First revaccination: 5 years after first dose<br>- Second revaccination: Nil<br>Route: Not mentioned<br>Dosage: Not mentioned<br>Storage: Not mentioned
		Smokers, newly diagnosed COPD, existing COPD, who have never received age-based dose	Non-indigenous: <65 years old		Timing:<br>-Initial dose: At diagnosis<br>-First revaccination: 5 years after first dose<br>- Second revaccination: at 65 years old or 5 years after second dose (whichever is later)<br>Route: Not mentioned<br>Dosage: Not mentioned<br>Storage: Not mentioned
			Indigenous: <50 years old		Timing:<br>-Initial dose: At diagnosis<br>-First revaccination: 5 years after first dose<br>- Second

					revaccination: at 50 years old or 5 years after second dose (whichever is later)<br>Route: Not mentioned<br>Dosage: Not mentioned<br>Storage: Not mentioned
		Smokers, newly diagnosed COPD, existing COPD, who have already received age-based dose	Non-indigenous: ≥65 years old		Timing:<br>-Initial dose: Nil<br>-First revaccination: 5 years after first dose<br>- Second revaccination: Nil<br>Route: Not mentioned<br>Dosage: Not mentioned<br>Storage: Not mentioned
			Indigenous: ≥50 years old		Timing:<br>-Initial dose: Nil<br>-First revaccination: 5 years after first dose<br>- Second revaccination: Nil<br>Route: Not mentioned<br>Dosage: Not mentioned<br>Storage: Not mentioned

Table S5. Summary of vaccination recommendation for Asthma.

Name of guidelines (Year of guideline publication), Country of origin of the guideline	Name of recommended vaccines, Strength of recommendation (strong/moderate/weak/not stated)	Details of recommendation for each vaccine		
		Specific indications (age, severity of disease, disease subgroup)	Specific contraindications and harms (side effects)	Vaccine administration (timing, route, dosage) and storage details

Global Initiative for Asthma (GINA): Global strategy for asthma management and prevention, update (2022), International	COVID-19, Strength of recommendation: Not stated	Asthma	Not mentioned	Not mentioned<br>Special consideration: first dose of biologic therapy and COVID-19 vaccine should not be given on the same day, to allow adverse effects of either to be more easily distinguished
	Influenza, Strength of recommendation: Not stated		Not mentioned	Timing: Annually<br>Route: Not mentioned<br>Dosage: Not mentioned<br>Storage: Not mentioned
	Pneumococcal, Strength of recommendation: Not stated		Not mentioned	Not mentioned
NHLBI and NAEPP: Expert panel report 3 – Guidelines for the diagnosis and management of asthma (2007), United States	Inactivated influenza vaccination, Strength of recommendation: Not stated	Asthma (children more than 6 months of age and adults)	Not mentioned	Not mentioned<br>Special consideration: - Should not be given with the expectation that it will reduce either the frequency or severity of asthma exacerbations during the influenza season<br><br>- patients who have documented histories of anaphylactic reactions after ingestion of egg protein and documented evidence of current allergic sensitization to eggs (skin testing or in vitro antigen-specific IgE antibody testing), the risk/benefit ratio of administration of influenza vaccine should be reviewed carefully
National Asthma Council Australia (NACA): Australian asthma handbook (2022), Australia-New Zealand (2022), Australia-New Zealand	Influenza, Strength of recommendation: Not stated	- patients with severe asthma, defined as those who need frequent hospital visits and multiple medicines for asthma<br>- all adults ≥65 years<br>- patients with COPD<br>- pregnant women<br>- for any adult who wishes to avoid influenza	Contraindications: person is receiving high-dose oral steroid therapy<br><br>Harms: Not mentioned	Timing: Annually<br>Route: Not mentioned<br>Dosage: Not mentioned<br>Storage: Not mentioned
	Pneumococcal, Strength of recommendation: Not stated			Not mentioned

Gastrointestinal- related diseases

Table S6. Summary of vaccination recommendation for Cirrhosis and Other Chronic Liver Diseases.

Name of guidelines (Year of guideline publication), Country of origin of the guideline	Name of recommended vaccines, Strength of recommendation (strong/moderate/weak/not stated)	Details of recommendation for each vaccine		
		Specific indications (age, severity of disease, disease subgroup)	Specific contraindications and harms (side effects)	Vaccine administration (timing, route, dosage) and storage details
American College of Gastroenterology (ACG): Acute-on-chronic liver failure clinical guidelines (2022), United States	Hepatitis A and B, Strength of recommendation: Not stated	Patients with Chronic Liver Disease	Not mentioned	Not mentioned
American College of Gastroenterology (ACG): Clinical guideline – Alcoholic liver disease (2018), United States	Hepatitis A and B, Strength of recommendation: Not stated	Patients with alcoholic cirrhosis	Not mentioned	Not mentioned
	Pneumococcal, Strength of recommendation: Not stated		Not mentioned	Not mentioned
	Influenza, Strength of recommendation: Not stated		Not mentioned	Not mentioned
American Association of Clinical Endocrinology (AACE): Clinical practice guideline for the diagnosis and management of nonalcoholic fatty liver disease in primary care and endocrinology clinical settings (2022), United States	Hepatitis A and B, Strength of recommendation: Not stated	Persons With Chronic Liver Disease	Not mentioned	Not mentioned
	Pneumococcal polysaccharide vaccine (PPSV23), Strength of recommendation: Not stated		Not mentioned	Not mentioned
	Influenza, Strength of recommendation: Not stated		Not mentioned	Not mentioned
	Tdap, Strength of recommendation: Not stated		Not mentioned	Not mentioned
	Zoster, Strength of recommendation: Not stated		Not mentioned	Not mentioned
	HPV, Strength of recommendation: Not stated		Not mentioned	Not mentioned
	MMR, Strength of recommendation: Not stated		Not mentioned	Not mentioned

	Varicella, Strength of recommendation: Not stated		Not mentioned	Not mentioned
	COVID-19, Strength of recommendation: Not stated		Not mentioned	Not mentioned

Table S7. Summary of vaccination recommendation for Colon and rectum cancer.

Name of guidelines (Year of guideline publication), Country of origin of the guideline	Name of recommended vaccines, Strength of recommendation (strong/moderate/weak/not stated)	Details of recommendation for each vaccine		
		Specific indications (age, severity of disease, disease subgroup)	Specific contraindications and harms (side effects)	Vaccine administration (timing, route, dosage) and storage details
Indian Council of Medical Research (ICMR): Consensus document for management of colorectal cancer (2014), India	Influenza, Strength of recommendation: Not stated	Patients with Colorectal cancer undergoing chemotherapy	Contraindications: Not mentioned<br>- Minor infection, in the absence of fever or systemic symptoms, is not itself a contraindication to vaccination<br><br>Harms: immunological response may be impaired by chemotherapy	Timing: -should be given before chemotherapy<br>-for live attenuated influenza vaccine should be given at the end of chemotherapy cycle when the degree of immunosuppression is at its lowest<br>-Immunization should be postponed if a patient is suffering from an acute illness<br>Route: Not mentioned<br>Dosage: Not mentioned<br>Storage: Not mentioned
	Hepatitis B, Strength of recommendation: Not stated			Timing: -should be given at the end of chemotherapy cycle when the degree of immunosuppression is at its lowest<br>-Immunization should be postponed if a patient is suffering from an acute illness<br>Route: Not mentioned<br>Dosage: Not mentioned<br>Storage: Not mentioned
	MMR, Strength of recommendation: Not stated		Contraindications: -Never be administered to immunocompromised patients, including those receiving chemotherapy, within 6 months after	Not mentioned<br>Special consideration: Immunization should be postponed if a patient is suffering from an acute illness

	BCG,<br>Strength of recommendation: Not stated		receiving chemotherapy<br>- Minor infection, in the absence of fever or systemic symptoms, is not itself a contraindication to vaccination<br><br>Harms: Not mentioned	Not mentioned<br>Special consideration: Immunization should be postponed if a patient is suffering from an acute illness
	Yellow Fever,<br>Strength of recommendation: Not stated			Not mentioned<br>Special consideration: Immunization should be postponed if a patient is suffering from an acute illness

Table S8. Summary of vaccination recommendation for Stomach Cancer.

Name of guidelines (Year of guideline publication), Country of origin of the guideline	Name of recommended vaccines, Strength of recommendation (strong/moderate/weak/not stated)	Details of recommendation for each vaccine		
		Specific indications (age, severity of disease, disease subgroup)	Specific contraindications and harms (side effects)	Vaccine administration (timing, route, dosage) and storage details
Family Practice Oncology Network (FPON): Clinical practice guidelines on upper gastrointestinal cancer (2016), Canada	Influenza,<br>Strength of recommendation: Not stated	For gastric lymphoma	Not mentioned	Timing: Yearly in autumn<br>Route: Not mentioned<br>Dosage: Not mentioned<br>Storage: Not mentioned
	Pneumococcal,<br>Strength of recommendation: Not stated	For gastric lymphoma	Not mentioned	Timing: To be given at least 2 weeks before initiation of anti-lymohoid cancer treatment, repeat again after 5 years later<br>Route: Not mentioned<br>Dosage: Not mentioned<br>Storage: Not mentioned<br>Special consideration: Delay until at least 6 months after completion of all lymphoid cancer treatment and any other immunosuppressive treatment
	Tetanus/ Diphtheria,<br>Strength of recommendation: Not stated	For gastric lymphoma	Not mentioned	Timing: Every 10 years<br>Route: Not mentioned<br>Dosage: Not mentioned<br>Storage: Not mentioned
	Meningococcal C Conjugate + Meningococcal Quadrivalent Conjugate (A,C,Y,W-135) + Haemophilus Influenzae Type b (HiB) Vaccine,<br>Strength of recommendation: Not stated	For gastric lymphoma	Not mentioned	Timing:<br>- If the spleen is to be or was removed or treated with radiation, give all 3 at least 2 weeks before splenectomy or if spleen is already removed, give as soon as possible after splenectomy.<br>- Repeat every 5 years<br><br>Route: Not mentioned

				Dosage: Not mentioned<br>Storage: Not mentioned
	Polio vaccine ,<br>Strength of recommendation: Not stated	For gastric lymphoma	Contraindication: Oral polio vaccine contraindicated in patients with lymphoid cancer<br><br>Harms: Not mentioned	Timing: Every 10 years<br>Route: Intramuscular<br>Dosage: Not mentioned<br>Storage: Not mentioned
	Varicella Zoster,<br>Strength of recommendation: Not stated	For gastric lymphoma	Not mentioned	Timing: At least 2 weeks before initiation of anti-lymphoid cancer treatment<br>Route: Intramuscular<br>Dosage: Not mentioned<br>Storage: Not mentioned<br>Special consideration: If cannot be given before, delay until 6 months after completion of all lymphoid cancer treatment/ immunosuppressive treatment
Indian Council of Medical Research (ICMR): Consensus document for management of gastric cancer (2014), India	Influenza ,<br>Strength of recommendation: Not stated	Not mentioned	Not mentioned	Not mentioned
	Haemophilus Influenza Type B,<br>Strength of recommendation: Not stated	If patient did not receive during infancy	Not mentioned	Not mentioned
	Meningococcal,<br>Strength of recommendation: Not stated	Patients > 2 years	Not mentioned	Timing: One-time vaccination<br>Route: Intramuscular<br>Dosage: Not mentioned<br>Storage: Not mentioned
	Pneumococcal ,<br>Strength of recommendation: Not stated	Patients > 2 years	Not mentioned	Timing: - Administered at least 2-3 weeks pre-operation<br>- Administer again 5 years after splenectomy<br>Route: Intramuscular<br>Dosage: Not mentioned<br>Storage: Not mentioned

Other diseases

Table S9. Summary of vaccination recommendation for Diabetes.

Name of guidelines (Year of guideline publication), Country of origin of the guideline	Name of recommended vaccines, Strength of recommendation (strong/moderate/weak/not stated)	Details of recommendation for each vaccine		
		Specific indications (age,severity of disease, disease subgroup)	Specific contraindications and harms (side effects)	Vaccine administration (timing, route, dosage) and storage details

IDF: Global guideline for managing older people with type 2 diabetes (2013, updated 2017), International	Seasonal influenza vaccine, Strength of recommendation: Not stated	All older people with diabetes	Not mentioned	Not mentioned
Diabetes Canada: Guidelines (2018), Canada	Influenza vaccine, Strength of recommendation: Not stated	People with Diabetes	Not mentioned	Timing: Annual<br>Route: Not mentioned<br>Dosage: Not mentioned<br>Storage: Not mentioned
Diabetes Canada: Guidelines (2018), Canada	Pneumococcal vaccine, Strength of recommendation: Not stated	Persons with diabetes aged 19 to 64 years	Not mentioned	Timing:<br>- Vaccinate Pneu-P-23 at diagnosis<br>- 1-time revaccination is recommended for those ≥65 years of age (if the original vaccine was given when they were <65 years of age)<br>Route: Not mentioned<br>Dosage: Not mentioned<br>Storage: Not mentioned
Diabetes Canada: Guidelines (2018), Canada	Pneumococcal vaccine, Strength of recommendation: Not stated	For people with diabetes ≥65 years or with an immunocompromising condition (e.g. end stage renal disease)	Not mentioned	Timing:<br>- Pneu-C-13 vaccine administered first<br>- followed at least 8 weeks later by Pneu-P-23 vaccine<br>Route: Not mentioned<br>Dosage: Not mentioned<br>Storage: Not mentioned
American Association of Clinical Endocrinology Clinical Practice Guideline: Developing a Diabetes Mellitus Comprehensive Care	Influenza vaccine: IIV4 or RIV4 or LAIV,<br>Strength of recommendation: Very strong	Persons with Diabetes who are ≥6 months old	Not mentioned	Timing: Annual<br>Route: Not mentioned<br>Dosage: Not mentioned<br>Storage: Not mentioned
American Association of Clinical Endocrinology Clinical Practice Guideline: Developing a Diabetes Mellitus Comprehensive Care	Pneumococcal<br><br>PCV15 and PCV20,	Persons with Diabetes aged 19-64 years old	Not mentioned	Timing:<br>-When PCV15 is used, PPSV23 should be

Plan—2022 Update	Strength of recommendation: Strong			administered at least 12 months following the dose of PCV15<br>-A minimum interval of 8 weeks may be used for adults with immunocompromising conditions<br>Route: Not mentioned<br>Dosage: Not mentioned<br>Storage: Not mentioned
		≥65 years old		Timing:<br>-For adults ≥ 65 years old and who have not previously received PCV or whose vaccination history is unknown, PCV15 or PCV20 should be administered.<br>-When PCV15 is used, it should be followed by a dose of PPSV23<br>Route: Not mentioned<br>Dosage: Not mentioned<br>Storage: Not mentioned
	Hepatitis B,<br>Strength of recommendation: Very strong	Persons with Diabetes ≤59 years	Not mentioned	Timing: At diagnosis<br>Route: Not mentioned<br>Dosage: Not mentioned<br>Storage: Not mentioned
	Hepatitis B,<br>Strength of recommendation: Not strong	Based on risk and quality of immune response for adults aged ≥60 y		Not mentioned
	Tdap,<br>Strength of recommendation: Not strong	-	Not mentioned	Timing: Every 10 years following completion of the primary series in routine childhood vaccination<br>Route: Not mentioned

				Dosage: Not mentioned<br>Storage: Not mentioned
	COVID-19,<br>Strength of recommendation: Strong	All persons per FDA approval or emergency use authorization	Not mentioned	Not mentioned
	Recombinant zoster vaccine (RZV),<br>Strength of recommendation: Very strong	All adults aged ≥50 years old	Not mentioned	Not mentioned
Joint British Diabetes Societies for Inpatient Care (JBDS-IP): Clinical guideline on inpatient care of the frail older adult with diabetes (2022), United Kingdom	Influenza vaccine,<br>Strength of recommendation: Not stated	Older frail individuals with diabetes living in the community	Not mentioned	Not mentioned
	Pneumococcal vaccine,<br>Strength of recommendation: Not stated	Older frail individuals with diabetes living in the community	Not mentioned	Not mentioned
	Herpes Zoster vaccine,<br>Strength of recommendation: Not stated	Older frail individuals with diabetes living in the community	Not mentioned	Not mentioned
Research Society for the Study of Diabetes in India (RSSDI)-Endocrine Society of India (ESI): Clinical practice recommendations for the management of type 2 diabetes mellitus (2020) , India	Quadrivalent influenza vaccine,<br>Strength of recommendation: Not stated	All patients with T2DM with age ≥6 months	Contraindications:<br>- Egg allergy<br><br>- Recent history of Guillain-Barre syndrome within six weeks of a previous influenza vaccination<br><br>- Febrile illness or any acute infection<br>Harms: Not mentioned	Timing: Annual<br>Route: Not mentioned<br>Dosage: Not mentioned<br>Storage: Not mentioned
	Pneumococcal vaccine (either PCV 13 with PPSV23 or only PPSV23):<br>13-valent pneumococcal conjugate vaccine (PCV13) followed by PPSV23,<br>Strength of recommendation: Not stated	DM patients >50 years	Contraindications:<br>- Hypersensitivity to the active substances or to any of the excipients of the vaccine<br><br>-Febrile illness or any acute infection<br>Harms: Not mentioned	Timing: PCV13 vaccination should be followed up with a dose of PPSV23 at least 1 year later<br>Route: Not mentioned<br>Dosage: Not mentioned<br>Storage: Not mentioned
	Only 23-valent pneumococcal polysaccharide vaccine (PPSV23),	All patients with T2DM	Contraindications:<br>- Hypersensitivity to the active substances or to any of the	Timing:<br>- ≤64 years old: receive PPSV23 at diagnosis<br>- ≥ 65 years old:

		Strength of recommendation: Not stated		excipients of the vaccine<br><br>-Febrile illness or any acute infection<br>Harms: Not mentioned	receive another PPSV23<br>- second dose of PPSV23 must be at least 5 years apart from the previous dose of PPSV<br>Route: Not mentioned<br>Dosage: Not mentioned<br>Storage: Not mentioned
	Hepatitis B Virus vaccine (3 dose series),<br>Strength of recommendation: Not stated		All unvaccinated patients with diabetes	Not mentioned	Not mentioned
	Td/Tap,<br>Strength of recommendation: Not stated		Diabetic patients	Not mentioned	Timing: Substitute one time dose of Tdap; then boost with<br>Td every 10 years<br>Route: Not mentioned<br>Dosage: Not mentioned<br>Storage: Not mentioned
	MMR (Measles mumps rubella),<br>Strength of recommendation: Not stated		Diabetic patients	Not mentioned	Timing: 1 or 2 doses 4 weeks interval<br>Route: Not mentioned<br>Dosage: Not mentioned<br>Storage: Not mentioned
	Varicella,<br>Strength of recommendation: Not stated		Diabetic patients	Not mentioned	Timing: 2 doses, at least 4 weeks apart<br>Route: Not mentioned<br>Dosage: Not mentioned<br>Storage: Not mentioned
	Zoster,<br>Strength of recommendation: Not stated		Diabetic patients	Not mentioned	Timing 1 dose at 60 years old<br>Route: Not mentioned<br>Dosage: Not mentioned<br>Storage: Not mentioned
	Hepatitis A,<br>Strength of recommendation: Not stated		Diabetic patients	Not mentioned	Timing: 2 doses at least 6 months apart<br>Route: Not

				mentioned<br>Dosage: Not mentioned<br>Storage: Not mentioned
	HPV (Human papillomavirus),<br>Strength of recommendation: Not stated	Females, diabetic patients	Not mentioned	Timing:<br>-3 doses through age 26 years<br>-The second dose should be administered 1-2 months after the first dose<br>-Third dose should be administered 6 months after the first dose (at least 24 weeks after the first dose<br>Route: Not mentioned<br>Dosage: Not mentioned<br>Storage: Not mentioned
		Male, diabetic patients	Not mentioned	Timing: 3 doses through age 21 years<br>Route: Not mentioned<br>Dosage: Not mentioned<br>Storage: Not mentioned
Royal Australian College of General Practitioners (RACGP): Management of type 2 diabetes – A handbook for general practice (2020, updated 2021), Australia-New Zealand	Influenza vaccine,<br>Strength of recommendation: Not stated	All patients with T2DM	Not mentioned	Timing: Annual<br>Route: Not mentioned<br>Dosage: Not mentioned<br>Storage: Not mentioned
	Diphtheria, tetanus, pertussis,<br>Strength of recommendation: Not stated	All patients with T2DM, aged ≥65 years if they have not had one in the past 10 years	Not mentioned	Not mentioned
	Hepatitis B,<br>Strength of recommendation: Not stated	T2DM patients who are travelling to hepatitis B–endemic areas	Not mentioned	Not mentioned
	Herpes zoster ,<br>Strength of recommendation: Not stated	T2DM patients age 70–79 years old	Not mentioned	Not mentioned
	Pneumococcal vaccine (23-valent pneumococcal	All adults with T2DM	Not mentioned	Timing:<br>- First dose at

	polysaccharide vaccine (23vPPV), Strength of recommendation: Not stated			around age 18 years, or at time of diagnosis of type 2 diabetes<br>- Second dose 5–10 years later<br>-Third dose at least five years later or at age 65 years, whichever is later.<br>Route: Not mentioned<br>Dosage: Not mentioned<br>Storage: Not mentioned
Japanese Diabetes Society (JDS): Japanese clinical practice guideline for diabetes 2019 (published 2020), Japan	Influenza vaccine, Strength of recommendation: Weak	Elderly patients with diabetes	Not mentioned	Not mentioned
	Pneumococcal vaccine Strength of recommendation: Weak	Elderly patients with diabetes	Not mentioned	Not mentioned

Table S10. Summary of vaccination recommendation for CKD.

Name of guidelines (Year of guideline publication), Country of origin of the guideline	Name of recommended vaccines, Strength of recommendation (strong/moderate/weak/not stated)	Details of recommendation for each vaccine		
		Specific indications (age, severity of disease, disease subgroup)	Specific contraindications and harms (side effects)	Vaccine administration (timing, route, dosage) and storage details
KDIGO: Clinical practice guideline for the prevention, diagnosis, evaluation, and treatment of hepatitis C in chronic kidney disease (2018), International	Hepatitis A & B Vaccines Strength of recommendation: Level 1 strength of recommendation	History of HCV infection (whether NAT-positive or not)	Not mentioned	Not mentioned
KDIGO: Clinical practice guideline for the evaluation and management of chronic kidney disease, 2012 (2013),International	Influenza, Strength of recommendation: Level 1 strength of recommendation	Adults with CKD	Not mentioned	Timing: Annual<br>Route: Not mentioned<br>Dosage: Not mentioned<br>Storage: Not mentioned
	Polyvalent Pneumococcal Vaccine, Strength of recommendation: Level 1	eGFR <30 ml/min/1.73 m^2 (GFR categories G4-G5) and those at high risk of pneumococcal	Not mentioned	Timing: Revaccination within 5 years<br>Route: Not mentioned<br>Dosage: Not mentioned

	strength of recommendation	infection (e.g., nephrotic syndrome, diabetes, or those receiving immunosuppression)		Storage: Not mentioned
	Hepatitis B, Strength of recommendation: Level 1 strength of recommendation	at high risk of progression of CKD and have GFR <30 ml/min/1.73 m^2 (GFR categories G4-G5)	Not mentioned	Not mentioned
Centers for Disease Control and Prevention (CDC): Guidelines for vaccinating kidney dialysis patients and patients with chronic kidney disease (2012, reviewed 2015), United States	DTaP/Tdap/Td, Strength of recommendation: Not stated	All adults	Not mentioned	Not mentioned
	Hepatitis B (Recombivax HB), Strength of recommendation: Not stated	- all susceptible chronic hemodialysis patients<br>- pre-end-stage renal disease patients before they become dialysis dependent<br>- ≥20 years old	Not mentioned	Timing: Need for booster doses should be assessed by annual testing for antibody to hepatitis B surface antigen (anti-HBs). A booster dose should be administered when anti-HBs levels decline to <10 mIU/mL<br><br>Route: Not mentioned<br>Dosage: administered in a 3 dose schedule (40 µg/mL)<br>Storage: Not mentioned
	Hepatitis B (two Engerix-B), Strength of recommendation: Not stated		Not mentioned	Timing: Need for booster doses should be assessed by annual testing for antibody to hepatitis B surface antigen (anti-HBs). A booster dose should be administered when anti-HBs levels decline to <10 mIU/mL<br><br>Route: Not mentioned<br>Dosage: a 4 dose schedule, 20ug [1.0 mL doses] administered in 1 or 2 injections<br>Storage: Not mentioned
	Influenza (TIV), Strength of recommendation: Not stated	all persons aged ≥6 months	Contraindications: Live, attenuated Influenza vaccine is contraindicated in CKD patients	Not mentioned

			Harms: Not mentioned	
	MMR,<br>Strength of recommendation: Not stated	All adults	Not mentioned	Not mentioned
	23-Valent Pneumococcal Polysaccharide Vaccine (PPSV23),<br>Strength of recommendation: Not stated	- all adults aged ≥65 years<br>- adults at high risk aged 19–64 years	Not mentioned	Timing: Revaccination within 5 years for persons with functional or anatomic asplenia and for immunocompromised persons<br>Route: Not mentioned<br>Dosage: Not mentioned<br>Storage: Not mentioned
	13-Valent Pneumococcal Conjugate Vaccine (PCV13) ,<br>Strength of recommendation: Not stated	adults aged ≥19 years with immunocompromising conditions (including those with chronic renal failure or nephrotic syndrome), functional or anatomic asplenia, cerebrospinal fluid (CSF) leaks, or cochlear implants	Not mentioned	Timing:<br>- receive a dose of PCV13 first, followed by a dose of PPSV23 at least 8 weeks later<br>- second PPSV23 dose is<br>recommended 5 years after the first PPSV23 dose for persons aged 19–64 years with functional<br>or anatomic asplenia and for persons with immunocompromising conditions<br>- those<br>who received PPSV23 before age 65 years for any indication should receive another dose of the<br>vaccine at age 65 years, or later if at least 5 years have elapsed since their previous PPSV23 dose<br>- adults aged ≥19 years with immunocompromising conditions, functional or anatomic asplenia, CSF leaks, or cochlear implants, who previously have received ≥1 doses of

				PPSV23 should be given a PCV13 dose ≥1 year after the last PPSV23 dose was received<br>- those who require additional doses of PPSV23, the first such dose should be given no sooner than 8 weeks after PCV13 and at least 5 years after the most recent dose of PPSV23<br><br>Route: Not mentioned<br>Dosage: Not mentioned<br>Storage: Not mentioned
	Varicella, Strength of recommendation: Not stated	All adults	Not mentioned	Not mentioned
Department of Veterans Affairs (VA)/Department of Defense (DoD): Clinical practice guideline for the management of chronic kidney disease in primary care, 3rd edition (2014), United States	Influenza, Strength of recommendation: Strong	CKD	Contraindications: Live vaccines, including nasal influenza (LAIV), may be contraindicated in patients with CKD and severe immunodeficiency including treatment with immunosuppressive agents<br><br>Harms: Not mentioned	Not mentioned
	Zoster /shingles, Strength of recommendation: Strong	CKD		
	Varicella , Strength of recommendation: Strong	CKD		
	MMR, Strength of recommendation: Strong	CKD		
	Tdap, Strength of recommendation: Strong	CKD		
	Pneumococcal polysaccharide vaccine (i.e., PCV 13 and PPSV23), Strength of recommendation: Strong	CKD	Not mentioned	Not mentioned
	Hepatitis B, Strength of recommendation: Strong	CKD	Not mentioned	Not mentioned
Kidney Health Australia (KHA): Chronic kidney disease (CKD)	Influenza, Strength of recommendation: Not stated	all people with diabetes and / or ESKD	Not mentioned	Not mentioned

management in general practice – Guidelines and clinical tips to help identify, manage and refer patients with CKD in your practice, 3rd edition (2015), Australia-New ZealandAustralia-New Zealand	Pneumococcal, Strength of recommendation: Not stated	all people with diabetes and / or ESKD	Not mentioned	Not mentioned
Japanese Society of Nephrology (JSN): Essential points from evidence-based clinical practice guideline for chronic kidney disease, 2018 (published 2019), Japan	Influenza, Strength of recommendation: Grade 2	CKD	Not mentioned	Not mentioned
	Pneumococcal, Strength of recommendation: Grade 2	CKD	Not mentioned	Not mentioned

Table S11. Summary of vaccination recommendation for Stroke.

Name of guidelines (Year of guideline publication), Country of origin of the guideline	Name of recommended vaccines, Strength of recommendation (strong/moderate/weak/not stated)	Details of recommendation for each vaccine		
		Specific indications (age, severity of disease, disease subgroup)	Specific contraindications and harms (side effects)	Vaccine administration (timing, route, dosage) and storage details
HSFC: Canadian stroke best practice recommendations on secondary prevention of stroke (2017), Canada	Influenza Strength of recommendation: Level B evidence from randomized controlled trials	- Especially in patients with pre-existing cardiovascular risk factors	Not mentioned	Not mentioned
AHA: Scientific statement on self-care for the prevention and management of cardiovascular disease and stroke (2017), United States	Influenza, Strength of recommendation: Not stated	Not mentioned	Not mentioned	Timing: Annual Route: Not mentioned Dosage: Not mentioned Storage: Not mentioned
AHA/ASA: Guidelines for the primary prevention of stroke (2014),United States	Influenza, Strength of recommendation: Moderate	Not mentioned	Not mentioned	Timing: Annual Route: Not mentioned Dosage: Not mentioned Storage: Not mentioned
